# The lighthouse at the end of the chromosome*

**DOI:** 10.12688/f1000research.6664.1

**Published:** 2015-12-11

**Authors:** Yahya Benslimane, Lea Harrington

**Affiliations:** 1 Department of Molecular Biology, University of Montreal, Institute for Research in Immunology and Cancer, Montreal, Quebec, Canada; 2 Department of Biochemistry, University of Montreal, Institute for Research in Immunology and Cancer, Montreal, Quebec, Canada; 3 Department of Medicine, University of Montreal, Institute for Research in Immunology and Cancer, Montreal, Quebec, Canada

**Keywords:** Fluorescence microscopy, telomeres, telomere elongation, telomere spatial organization, time-lapse microscopy, single-molecule fluorescence, chromosome

## Abstract

Fluorescence microscopy can be used to assess the dynamic localization and intensity of single entities *in vitro* or in living cells. It has been applied with aplomb to many different cellular processes and has significantly enlightened our understanding of the heterogeneity and complexity of biological systems. Recently, high-resolution fluorescence microscopy has been brought to bear on telomeres, leading to new insights into telomere spatial organization and accessibility, and into the mechanistic nuances of telomere elongation. We provide a snapshot of some of these recent advances with a focus on mammalian systems, and show how three-dimensional, time-lapse microscopy and single-molecule fluorescence shine a new light on the end of the chromosome.

## Introduction

Oskar Heimstädt, who built the first fluorescence microscope, ended his 1911 paper with the following perspective: “If and to what degree fluorescence microscopy will widen the possibilities of microscopic imaging only the future will show” ^1^. More than a century later, fluorescence microscopy has proven transformative in our ability to illuminate almost all aspects of cellular biology. One of the more recent frontiers in fluorescence microscopy is the resolution of biological phenomena at the single molecule level, called single molecule fluorescence ^2^. Biological processes have evolved to be inherently heterogeneous, transient and dynamic ^3^, and therefore difficult to track. Single molecule microscopy often permits a birds-eye view of ephemeral and complex mechanisms. This review will focus on selected recent advances in high-resolution microscopy including, but not limited to, single-molecule fluorescence microscopy, that have enlightened our understanding of chromosome ends and the enzyme that replenishes them.

**Figure 1.**
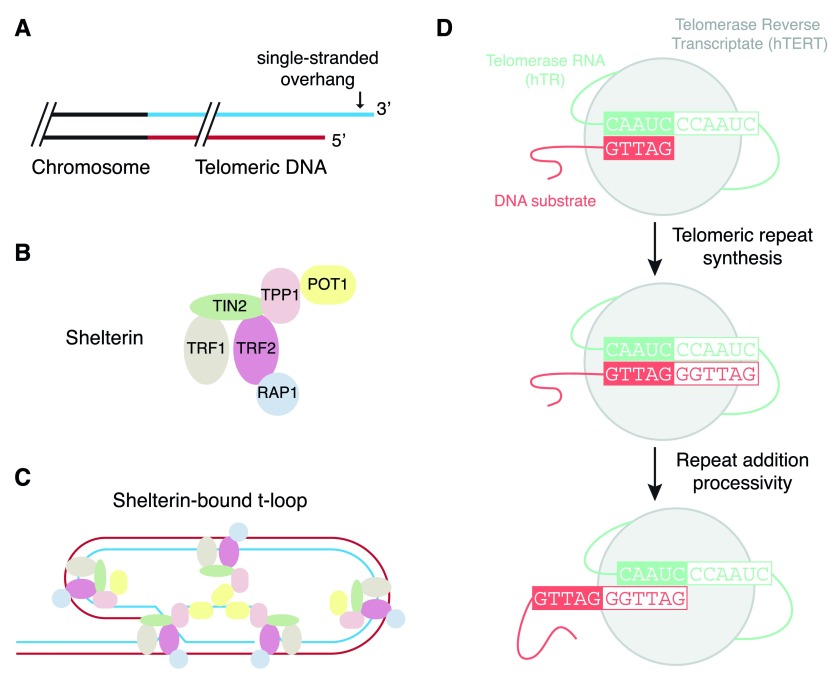
An overview of telomeres and telomerase. ( **A**) In many organisms, chromosome ends terminate in a single-stranded, G-rich overhang preceded by up to several kilobase pairs of double-stranded G-rich DNA. ( **B**) Telomeres are capped by a six-subunit complex called shelterin. ( **C**) Shelterin (particularly TRF2) promotes the formation of a higher order telomeric loop (T-loop) structure that serves to mask telomeres from the deleterious fates associated with a free DNA end ^5, 6^. ( **D**) The catalytic cycle of the core telomerase enzyme, comprised of a protein (TERT) and RNA (hTR) ^7^.

Many organisms must contend with the vulnerability of linear chromosome ends to enzymes that degrade, rearrange, or incompletely replicate DNA. This susceptibility to breakage, and their distinct “knob-like” appearance under the light microscope, made them an early target of study by scientists such as Barbara McClintock and Hermann Müller ^4^. We now appreciate that telomeres are a highly specialized nucleoprotein structure whose maintenance is critical to genome stability ( Figure 1A–C) ^5, 6^. Replenishment of the G-rich sequences that comprise the telomeres is carried out by telomerase, whose core components are a reverse transcriptase and an integral RNA that provides the telomere template ( Figure 1D) ^7^.

## Zooming in on telomeres

Fluorescence microscopy has revolutionized our ability to probe the length, location, and recombination of telomeres *in vivo* through the application of fluorescently labeled peptide nucleic acids that bind tightly and specifically to telomeric DNA ^8– 10^. These techniques have uncovered interesting distinctions in the three-dimensional (3D) localization of mammalian telomeres in normal cells *versus* cancer cells ^11, 12^. Time-lapse microscopy has also revealed increased mobility of telomeres upon induction of a DNA break ^13^ as well as unexpected long-range telomere associations in telomerase-negative cells after DNA breakage ^14^. In the budding yeast *Saccharomyces cerevisiae*, fluorescent tagging of several components of telomerase have also provided considerable insight into the temporal and spatial dynamics of telomerase recruitment in living cells ^15– 18^.

**Figure 2.**
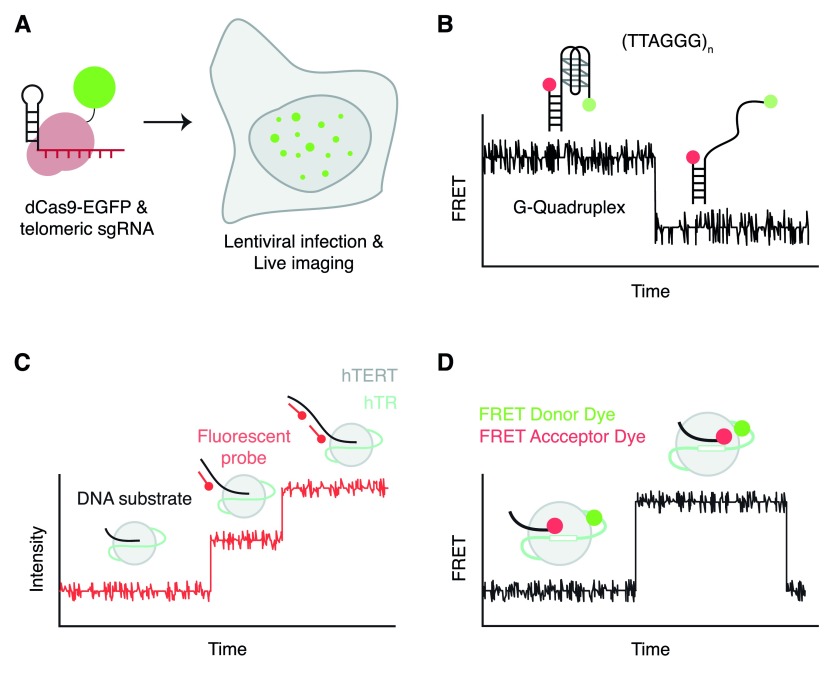
Selected recent advances in single-molecule microscopy that illuminate telomeres and telomerase. ( **A**) The use of a fluorescently tagged Cas9 and a guide RNA specific to telomeres to measure telomere dynamics and length in live cells ^20^. ( **B**) The ability of single-molecule Förster resonance energy transfer (smFRET) to measure the dynamics of G-quadruplex folding ^27, 30– 36, 38^. ( **C**) The application of fluorescent probes complementary to telomeric substrates to measure the elongation properties of telomerase ^47^. ( **D**) The application of FRET to assess the intermolecular proximity of the DNA substrate and the RNA subunit of telomerase during telomere synthesis ^48^.

Fluorescence microscopy is also being combined with other leading-edge technologies to directly target specific genomic regions, including the telomere. Using the CRISPR/Cas system in which Cas9 can be guided to specific genomic loci using a small RNA ^19^, Chen and colleagues targeted an enhanced green fluorescent protein (EGFP)-tagged catalytically inert Cas9 specifically to telomeres ^20^. Telomere-recruited Cas9 demonstrated a punctate pattern that spatially overlapped with that of the telomeric DNA binding protein, telomeric-repeat binding factor 2 (TRF2). Furthermore, EGFP intensity correlated linearly with telomere intensities obtained using fluorescence *in situ* hybridization (FISH) ( Figure 2A) ^20^. This method could also be employed to visualize Cas9 directed to a single genomic locus ^20^. Technological advances such as these may soon permit the ability to explore the spatial and dynamic localization of telomeres and telomerase in living mammalian cells to a similar extent as has been explored in budding yeast.

The telomere terminus ends in a 3’ G-rich overhang of variable length that can invade the upstream double-stranded telomeric DNA to form a structure called a T-loop ^21^. Super-resolution microscopy, specifically stochastic optical reconstruction microscopy (STORM), has recently enabled an unprecedented visualization of T-loops that are crosslinked and purified from murine cells ^22^ ( Figure 1B,C). The loss of T-loops occurred specifically upon loss of the shelterin subunit TRF2, and not other shelterin components, which provides an elegant demonstration that it is the T-loop structure that protects ends from the deleterious molecular events observed when TRF2 is removed from telomeres ^22, 23^.

Telomeric DNA sequences can also form *in vitro* intra- or inter-parallel structures called G-quadruplexes, and evidence is accruing to suggest their existence at telomeres and at interstitial G-rich regions ^24^. Single-molecule *Förster* resonance energy transfer (smFRET) has been applied to the molecular dynamics of G-quadruplex formation *in vitro* ^25– 28^ ( Figure 2B). The smFRET approach has several advantages that permit a high-resolution view of G-quadruplex dynamics. Firstly, this technique can resolve different conformations using FRET signal intensities and Gaussian curve fitting ^29^. Secondly, individual single-molecule traces reveal dynamic switching between states in real time. Hidden Markov modeling can then extract the dwell time of each molecule, which is a reflection of the stability of each state ^29^. Applying smFRET to the analysis of shelterin binding to telomeric substrates *in vitro*, several groups have investigated the intricate relationship between G-quadruplexes and POT1/TPP1 (protection of telomere 1/Pot1-interacting protein TINT1-PTOP-PIP1) ^30, 31^. Furthermore, this technique also revealed the binding and unfolding of G-quadruplexes by proteins such as RAD51, WRN, BLM, RecQ and RPA ^32– 36^. Recently, the use of smFRET combined with magnetic tweezers spectroscopy ^37^ has been used to measure the thermodynamic properties of G-quadruplex folding ^38^. These findings provide important insights into the dynamics of telomeric DNA structure in a more native nucleoprotein context.

## Shining a light on telomerase

The telomerase reverse transcriptase, TERT, is able to synthesize new telomeric DNA, one nucleotide at a time, by virtue of an integral telomerase RNA that contains a short telomere-complementary sequence ( Figure 1D). Although TERT shares several features in common with other viral reverse transcriptases, one unique aspect is its ability to repeatedly copy the same template for many cycles in an iterative process termed repeat addition processivity (RAP) ^39, 40^. Although much information has been gleaned using standard biochemical techniques regarding the complex DNA-protein, RNA-protein and protein-protein interactions that contribute to telomerase RAP (for a few current examples, see 41– 46), it is only recently that single-molecule fluorescence has been brought to bear on the elongation properties of telomerase *in vitro* ^47^. Hwang and colleagues immobilized immunopurified telomerase from cell extracts on a surface and employed a fluorescently labeled probe complementary to telomeric DNA to obtain a digital readout of telomerase activity in real time ( Figure 2C). Their results suggested that active telomerase can exist in two dynamic states defined as an initial activation period followed by an extension period in which telomere elongation was visualized as a step-wise increase in fluorescence ^47^. In addition, they found that TPP1-POT1 stimulated the elongation rate and overall product length ^47^, consistent with previously described properties of TPP1-POT1. Although the nature of the fluorescent probe binding (which comprises the sequence 5′-CCCTAACCCTAACCC-3′) precludes single base resolution, this technique promises to provide an unprecedented, single-molecule view of the intricacies of the telomerase elongation cycle.

In another smFRET approach, the distance between the region 5’ of the RNA template of telomerase and a DNA substrate was followed during telomeric repeat synthesis ( Figure 2D) ^48^. Using different combinations of regular and chain-terminating nucleotides, information about the position of the DNA oligonucleotide substrate was registered at single-base pair resolution during elongation. While the substrate appears in a compact conformation after initial binding by telomerase, after a round of DNA synthesis the substrate realigns with the template *via* Watson-Crick base pair interactions. The aforementioned study by Parks and colleagues suggests that DNA dissociation and realignment is not the limiting step, and that an additional conformational adjustment is necessary after DNA:RNA repositioning to re-acquire a catalytically competent state ^48^. This study illustrates the power of non-linear Gaussian curve fitting and hidden Markov modeling to extract the dwell time of the different FRET states from individual traces, which in turn permits a very precise dissection of the catalytic mechanism, albeit only over the short distances in which FRET can be observed.

smFRET has also been used to evince the real-time dynamics of folding of the pseudoknot domain with the telomerase RNA ^49– 51^. It has also been applied to examine the assembly and activity of the *Tetrahymena thermophila* telomerase RNP ^51– 53^ and, more recently, the role of the N-terminal domain of *T. thermophila* TERT in the stabilization of short RNA:DNA hybrids during telomerase catalysis ^54^. In addition to valuable insights into telomerase catalysis, these techniques might also permit a precise elucidation of the mechanism-of-action of chemical modulators of telomerase activity, as well as an *in vivo* determination of human telomerase component stoichiometry, as was recently demonstrated for budding yeast telomerase ^18^.

## Future perspectives

In the year in which fluorescence microscopy was first described, Arthur Brisbane offered the sage advice: “Use a picture. It’s worth a thousand words.” ^55^. We have reached a technological watershed in biology that will enable an unprecedented single-molecule and high-resolution view of the inner workings of many cellular machines. As we have illustrated here with but a few selected examples, fluorescence microscopy can be applied in many different ways to different problems, but key advances are the ability to dissect individual events instead of ensemble, population-based outputs, and to permit dynamic measurements in living cells, in real time. How far we have come from seeing telomeres as a cytogenetic “knob”, and how far we have yet to come.
